# Chronic within-hive video recordings detect altered nursing behaviour and retarded larval development of neonicotinoid treated honey bees

**DOI:** 10.1038/s41598-020-65425-y

**Published:** 2020-05-26

**Authors:** Paul Siefert, Rudra Hota, Visvanathan Ramesh, Bernd Grünewald

**Affiliations:** 1 Institut für Bienenkunde, Polytechnische Gesellschaft Frankfurt am Main, Goethe-Universität, Frankfurt am Main, Germany; 2 Center for Cognition and Computation, Institut für Informatik, Goethe-Universität, Frankfurt am Main, Germany

**Keywords:** Behavioural methods, Social behaviour, Agroecology, Behavioural ecology, Developmental biology

## Abstract

Risk evaluations for agricultural chemicals are necessary to preserve healthy populations of honey bee colonies. Field studies on whole colonies are limited in behavioural research, while results from lab studies allow only restricted conclusions on whole colony impacts. Methods for automated long-term investigations of behaviours within comb cells, such as brood care, were hitherto missing. In the present study, we demonstrate an innovative video method that enables within-cell analysis in honey bee (*Apis mellifera*) observation hives to detect chronic sublethal neonicotinoid effects of clothianidin (1 and 10 ppb) and thiacloprid (200 ppb) on worker behaviour and development. In May and June, colonies which were fed 10 ppb clothianidin and 200 ppb thiacloprid in syrup over three weeks showed reduced feeding visits and duration throughout various larval development days (LDDs). On LDD 6 (capping day) total feeding duration did not differ between treatments. Behavioural adaptation was exhibited by nurses in the treatment groups in response to retarded larval development by increasing the overall feeding timespan. Using our machine learning algorithm, we demonstrate a novel method for detecting behaviours in an intact hive that can be applied in a versatile manner to conduct impact analyses of chemicals, pests and other stressors.

## Introduction

Analyses of agrochemical effects, such as neonicotinoids, are necessary to preserve healthy populations of honey bee colonies. According to the latest risk assessment of the European Food Safety Authority (EFSA 2018^1^), screening 735 documents that monitored the effects of the neonicotinoids clothianidin, imidacloprid and thiamethoxam, such experiments are most commonly based on the behaviour or vitality of caged bees studied in a laboratory (314 experiments; cf.^2^; e.g.^3^). Other approaches comprise colony, field, or semi-field experiments (74, 101 and 27 experiments, respectively). While field studies have limitations in analysing individual behaviour, laboratory studies typically allow only restricted conclusions on colony conditions (cf.^4^).

Various effects of neonicotinoids on honey bee behaviour have been elucidated in the past, including impaired learning and memory^5,6^, influence on motor function^7^, and impaired navigation and foraging behaviour^8–11^. Furthermore, effects on immune system^12,13^ and brood viability^14^ have been also demonstrated, though the field relevance of these impairments remains unclear^15^. On the other hand, field studies involving the placement of bee hives close to treated crop fields or feeding full hives with neonicotinoids in the field failed to show homogenous systematic effects on colony fitness^16–21^ (reviews^22,23^).

Notably, the effects of neonicotinoids on brood development are controversial. Retarded brood development has been demonstrated in various *in vitro* rearing experiments (for review see^24^), but some authors summarize no delaying effect of clothianidin on honey bee larvae^25^ or on the solitary bee *Osmia bicornis*^26^ under such conditions. While some authors have reported the relatively high larval toxicity of neonicotinoids through *in vitro* experiments (cf.^14^), others describe a relatively high tolerance when using combs in functioning hives^27^. This suggests that hives with many individuals may compensate adverse effects or present *in vitro* diets and rearing experiments requiring improvement since a universal consensus on a specific methodology for testing pesticides on honey bee brood has yet to be achieved^14^.

Evidence shows that neonicotinoids impact the size of food producing glands^28,29^ and their non-neuronal acetylcholine secretion^30^, and reduce the amount of protein in workers^31^. Since a relatively high concentration of non-neuronal acetylcholine in brood food suggests its crucial role in development^32–34^, neonicotinoids, that act agonistically on the acetylcholine receptor, are likely to interfere with developmental processes (for review see^24^). It was recently demonstrated that *in vitro* larval development is impaired upon blockage of acetylcholine receptors and that acetylcholine is synthesized in the canal cells of hypopharyngeal glands^30^. However, the target within developing larvae and mode of action of neonicotinoids within larvae remain to be determined.

To understand the social organisation of insect colonies, including those of honey bees, cooperative brood care is a common research focus^35^. However, cooperative brood care remains rather difficult to observe in a living colony because visual inspections must be performed deep within the colonies. There is a current lack of methods available to monitor individual behaviours involved in cooperative brood care, while changes in larval nutrition, which depend on age and caste, make this a complex field in honey bee research. In this report, we describe a novel video-based technique for recording and analysing brood care within honey bee colonies and studying the effects of insecticides on brood care behaviour in living hives. Our approach overcomes the existing gap between laboratory and field effects by providing non-invasive long-term quantifications of within-hive brood care. We monitored larval development and larvae-worker interactions in longitudinal truncated brood cells in undisturbed small observation hives and developed an analysis method to determine the cause, number and duration of brood cell visits over several weeks of continuous recording time. For the very first time, we report that field realistic concentrations (cf.^17,31,36–38^) of neonicotinoids alter nursing behaviour within honey bee colonies.

## Results

### Colony Development

**Figure 1:**
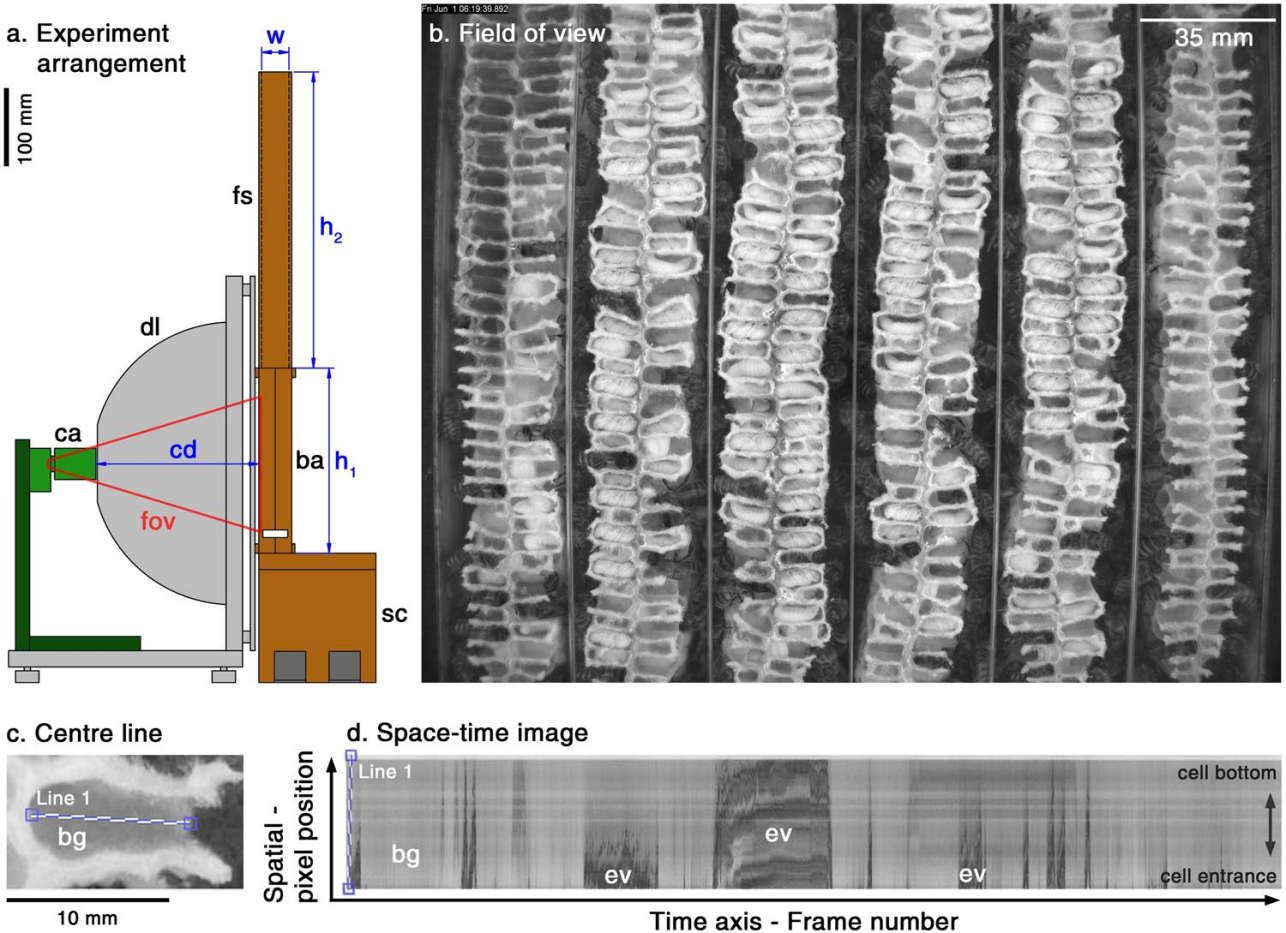
Recording and analysis methods to detect nursing parameters within cells. In our experiment arrangement (**a**) we recorded longitudinal truncated cells in the brood area (ba; h1 = 235 mm) of the observation hive using a digital video camera (ca) at focal distance (cd = 210 mm) and red light-emitting (λ_peak_ = 660 nm) dome-lighting (dl). The observation hive was 35 mm in width (w) and fitted with two MiniPlus-frames in the food super (fs, h2 = 380 mm) and a feeder on top (not shown). To make the brood area level with the lighting and camera, the hive was fixed on a sub-construction (sc, height: 165 mm). The field of view (fov; **b**) covered 220 × 175 mm and approximately 420 truncated cells (13.5% of total brood area). Cell visits were visualised by concatenating the temporal change of each pixel of a line (Line 1; **c**) along the x-axis, thereby generating a space-time image (**d**) with the cell entrance at the bottom. Since bees appeared as dark structural changes (‘events’; ev) in front of the background (bg in **c** and **d**), event parameters were automatically determined after applying a user defined grey-threshold for event detection. Thereafter, feeding events were either manually classified, or – later, after development – automatically classified using a convolutional neural network algorithm analysing the event structure (e.g. feeding event structure looked similar to the one shown in the centre of the image).

During the first few days after experiment initiation workers used existing wax of the provided combs to fix them to the glass of the brood area (experimental design in Fig. 1). This wax was primarily taken from the visible (first) cell layer so that these cells appeared shrinking during this period (see Supplementary Video S1). First layer cells were gradually extended in the following days (see Supplementary Videos S1 and S2). Eggs were visible in the first layer shortly after experiment initiation but nurses did not continue feeding hatched larvae and cannibalized them (as described for other larvae^39^) until the inner cells were occupied to a certain degree (most of inner cells were invisible). Since cell walls were very thin developing larvae were visible in the second cell layer as well. Rearing in the second layer preceded rearing larvae in the first, showing that nurses prioritised the inner cells for brood rearing (see Supplementary Video S2). Successful brood rearing (development until capping) in the first layer did not start before five to seven days into the experiment so we delayed recordings likewise. Therefore, all colony members were exposed to treatments for at least this period of time prior to experiment analysis. In the first cell layer, offspring development experienced a peak in May and June. Thereafter, the number of occupied brood cells decreased and displayed scattered, if any, capped cells during August. Therefore, the possible time window for successful experiments was constrained to May to July.

### Characterization of behaviours

Our experiment equipment allowed us to gather an extensive amount of behavioural data of workers within the cells. We manually distinguished behaviours to detect feedings within the cells and present characteristics of the most commonly seen behaviours: building, feeding and a motionless state with indications for heating behaviour (see below). During building activity workers went back and forth within the cell several times with frequent antennal movement, head movement, and longitudinal turns.

Feeding events were separated into two distinct behaviours since feedings were always preceded by an inspection. Inspections prior to food supply were discernible by workers entering the cell with strong antennal movements and mandibles and antennal tips pointing towards the larva, sometimes accompanied by longitudinal turns. The turns occurred more often in cells containing young larvae, reflecting the workers attempting to locate the correct position for food provision. Inspection duration varied according to larval age. In cells of young larvae inspections took relatively long compared to the feeding duration. Inspections of five-day old larvae were sometimes reduced to an undetectable minimum at the given frame rate. Food provision was initiated by clattering mandibles (seen in high frame rate macro-recordings) during which the worker gradually approached until secreting food next to the larva. During food provision nurses remained motionless within the cell. The gradual approach followed by an entire stop of body motion was an important characteristic in analysis. Throughout inspections and feedings head alignment of the worker (pointing towards the larva) with consistent antennal movement were other well detectable characteristics. Secreted jelly was only visible if the feeding location was at or near the glass. Furthermore, worker jelly could not be distinguished from modified worker jelly (see^40^; distinction to royal jelly see^41^). However, based on the described worker appearance and frequent larval movement during food provision, feedings were very easily detected.

Presumable heating events were discriminated by a head alignment (not towards the larva) and little to no antennal movement. We observed up to 1.5 hours of continuous long-term cell occupations displaying almost solely abdominal movements, if any. Although we could not measure emitted heat, we found supporting evidence that the events represent heating. In a fifth experimental arrangement used for general macro-recordings that was located on top of a heat-emitting radiator, those continuous long-term cell occupations were not present; instead, workers supplied a clear fluid inside the cell that was soon taken up again, which we interpreted as cooling (see Supplementary Video S3). Vice versa, cooling events were absent from the 16 analysed experiments of this study, which were not located directly over a heat-emitting radiator. Primarily during this heating activity, workers pushed eggs down, thus displaying the main reason for egg descent. This is supported by missing egg descent observations for hives placed above heat-emitting radiators, where heating events were not present (cf. abruptly moving eggs in Supplementary Video S2 and missing decent in Supplementary Video S3).

### Nursing parameters

**Figure 2:**
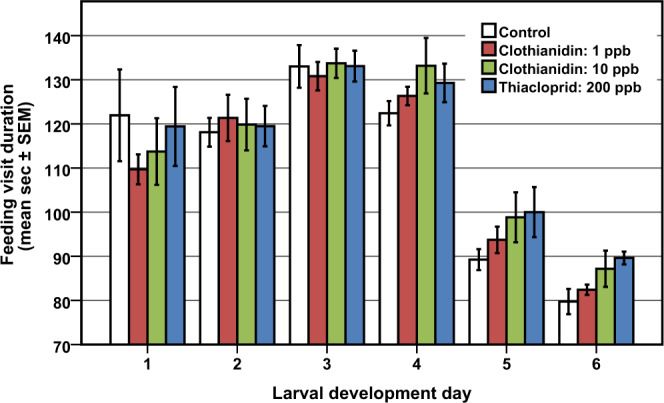
Duration of feeding visits on larval development days. During the first two days mean feeding visits lasted about 2 minutes in control colonies, and took the longest on the third day. On day five feeding visit duration majorly decreased to 1.5 minutes. See text for details. Treatments induced a reoccurring dose-dependent effect on days 4 to 6 of development, which was not significant due to differing results in one of the four experiments (n = 4; cf. Figs. 2, 3). The feeding visit duration includes inspection times prior to feedings. SEM = standard error of mean.

**Figure 3:**
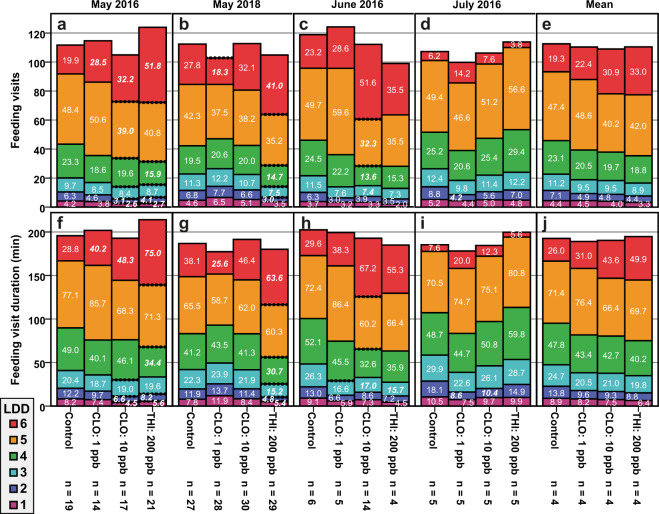
Feeding visits and durations in all experiments. Numbers in boxes represent the mean value on each larval development day (LDD; colour-coded) for feeding visits (**a**–**b**) and feeding duration (**f**–**j**). Bold italic numbers in boxes represent statistical differences to the control on this LDD (Bonferroni-Holm type I error-corrected adjusted *p* of Student’s *t*-test after significant one-way ANOVA; number of tests used for *p*-value adjustment = 3). The upper ends of the coloured boxes show the mean cumulative value on the y-axis, equivalent to the sum of all numbers below. If dotted, significant statistical differences (same test) of cumulative values to the control were present. Graphs (**e**,**j)** show the mean of (**a**–**d** and **f**–**i)**, respectively. Treatments were in most cases below the controls daily and cumulative and value, but levelled throughout LDD 6 (red boxes). The upper ends of LDD 6 represent the total value until cell capping. Feeding duration was strongly correlated to the number of feeding visits. Parameter means of all experiments (Mean) were not significant due to the results of July 2016. Abbreviations: CLO = clothianidin; THI = thiacloprid; ppb = parts per billion; SEM = standard error of mean. Samples sizes (n) are number of analysed cells in the experiments (**a,f**: May 2016, **b,g**: May 2018, **c,h**: June 2016, **d,i**: July 2016) and the number of colonies (**e,j**: Mean).

**Figure 4:**
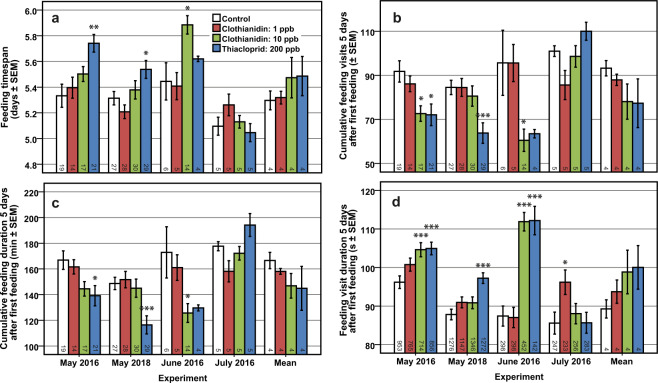
Feeding timespan and nursing parameters on larval development day 5. Mean feeding timespan (**a**) from the first to the last feeding was significantly increased up to 0.44 days (10.56 hours) after chronic treatment with 10 ppb clothianidin or 200 ppb thiacloprid in all experiments except July 2016. Five days after first feeding, neonicotinoid treatment significantly reduced mean cumulative feeding visits (**b**) and mean cumulative feeding duration (**c**), whereas mean feeding visit duration was prolonged (**d**). This shows an interdependence of the affected nursing parameters and feeding timespan (or development time of the larvae). Asterisks indicate significant differences to control (Bonferroni-Holm adjusted *p*-value of Student’s *t*-test: * <0.05, ** <0.01 and *** <0.001 levels; number of tests used for *p*-value adjustment = 3). Sample sizes at lower end of bars represent the analysed cells within experiments (May 2016, May 2018, June 2016, July 2016) or the number of colonies within the mean (Mean).

**Figure 5:**
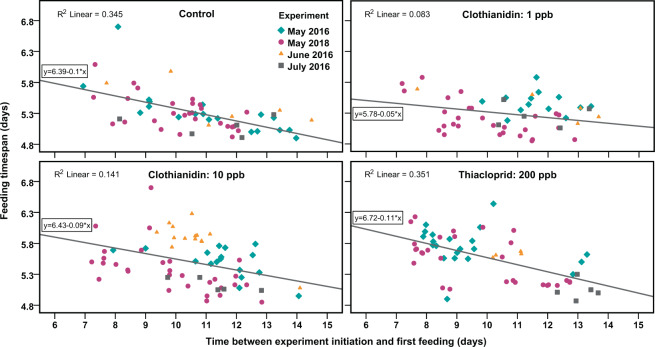
Feeding timespan of larvae in dependence of time passed between experiment initiation and the first feeding. Each data point represents the feeding timespan of a larva within the brood area in the respective treatment (graph title) of an experiment (symbols). Feeding timespan was significantly shortened by the time passed in all treatments (One-way ANOVA) but homogeneity of regression slopes was preserved (see text). Therefore, we found no evidence that a time cumulative effect of the neonicotinoids was present over the measured timespan from 7 to 20 days (20 days includes 14 days between experiment initiation and first feeding and subsequent larval development time).

**Figure 6:**
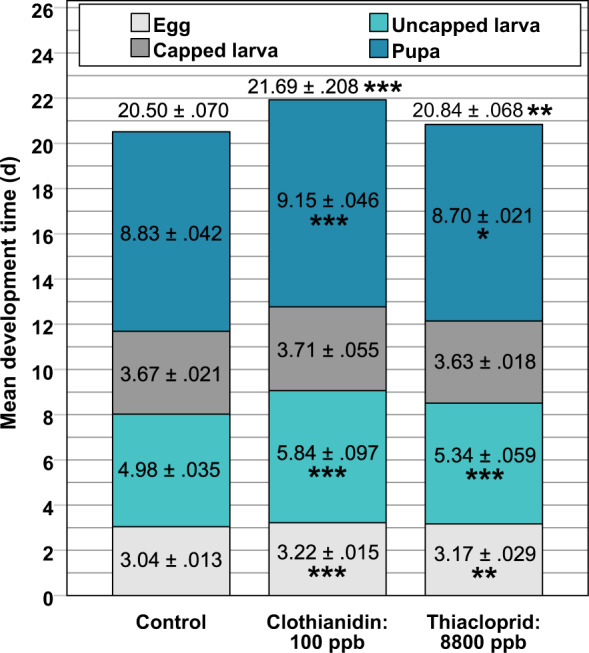
Worker development times (mean ± SEM) of various observable stages in high neonicotinoid treatments. The embryonic (egg) stage was significantly delayed in colonies treated with 100 ppb clothianidin and 8800 ppb thiacloprid. Greatest relative delay was present in the uncapped larval stage (17.3% and 7.2% increase in clothianidin and thiacloprid treatments, respectively). No significant differences were observed during larval development in the capped cell. While clothianidin increased pupal development time, thiacloprid surprisingly decreased it. The control needed 20.50 ± 0.07 days for complete worker ontogenesis (numbers for complete ontogenesis are not the sum of means). In the 100 ppb clothianidin treatment complete ontogenesis was delayed by 1.19 days or 28.56 hours and in the 8800 ppb thiacloprid treatment by 0.34 days or 8.16 hours. Statistical test: ANOVA, Bonferroni-Holm adjusted *p*-values of Student’s *t*-test; number of tests used for *p*-value adjustment = 2. Asterisks indicate significance levels: * <0.05, ** <0.01 and *** <0.001. Number of analysed cells per colony in order of appearance: 8, 13, 10 of one colony between May and July 2017. SEM = standard error of mean.

To test whether neonicotinoids influenced feeding behaviour in the colonies, we determined the duration of each feeding visit (feeding visit duration; Fig. 2) and subsequently calculated the following nursing parameters for each larval development day (LDD): the daily and cumulative feeding visits (feeding visits/cumulative feeding visits; Fig. 3a–e) and duration (feeding duration/cumulative feeding duration; Fig. 3f–j). We also analysed the feeding timespan of the nurses, indicating the larval development time (Fig. 4a). Furthermore, we show the effects on LDD 5 for cumulative feeding visits (Fig. 4b) and duration (Fig. 4c), and feeding visit duration (Fig. 4d). Finally, we tested if feeding timespan was depending on the duration of exposition to the substances (Fig. 5), and tested the influence on development after high neonicotinoid administration (Fig. 6). By statistically analysing the results for each experiment individually, in addition to the mean, we allow a transparent view of significant differences within each colony.

We analysed the time spent in the cell during a feedings visit shown in Fig. 2. This includes inspection times prior to feedings. On LDDs 1 and 2 feeding visits lasted 122.0 ± 10.4 (mean ± SEM; n = 4) and 118.1 ± 3.3 seconds, respectively, in control colonies. We observed relatively high variations on LDD 1 due to varying time for proper inspection of the location of the newly hatched larva. Nurses in all treatments fed the longest on LDD 3 (133.0 ± 4.8 seconds in control) followed by LDD 4 (122.4 ± 2.8 seconds in control). On LDD 5, a 25% decrease in feeding visit duration was present in all treatments (89.3 ± 2.4 seconds in control). This possibly represents the change in nutrition from worker jelly to modified worker jelly, when increasingly honey stomach contents are fed. On LDD 6 feeding visit duration was the shortest (79.8 ± 2.8 seconds in control). As already graspable on LDD 4, we observed a dose-dependent appearance on LDD 5 and 6 showing that treated bees visited the cells longer compared to the control. Neonicotinoid treatment showed significant differences for feeding visit duration on LDD 5 (Fig. 4d) in May 2016 (*F*(3, 3284) = 6.22, *p* < 0.001), May 2018 (*F*(3, 5037) = 7.74, *p* < 0.001), June 2016 (*F*(3, 1186) = 26.98, *p* < 0.001) and July 2016 (*F*(3, 1015) = 2.96, *p* = 0.031), but not the mean (*F*(3, 12) = 1.25, *p* = 0.336). Feeding visit duration was significantly increased in 10 ppb clothianidin treated colonies in May 2016 (t = −3.412, *p* = 0.001, n = 714) and June 2016 (t = −6.720, *p* < 0.001, n = 452), and 200 ppb thiacloprid treatment increased feeding visit duration in May 2016 (t = −3.763, *p* < 0.001, n = 856), May 2018 (t = −4.840, *p* < 0.001, n = 1272) and June 2016 (t = −5.439, *p* < 0.001, n = 142). 1 ppb clothianidin significantly increased feeding visit duration in July 2016 (t = −2.495, *p* = 0.039, n = 233). From a total of 16 post-hoc comparisons of feeding visit duration on LDD 5, 10 were not significant.

The number of feeding visits per day increased from LDDs 1 to 5 in control colonies. We counted 4.4 ± 0.3 feeding visits (mean ± SEM, n = 4) on the first and 7.1 ± 0.6 feedings on the second day, followed by 11.2 ± 0.6, 23.1 ± 1.3 and 47.4 ± 1.7 average feedings on the third, fourth and fifth day, respectively (Fig. 3e; numbers in coloured boxes; overview in Supplementary Table S1). On LDD 6, larvae received an average of 19.3 ± 4.7 feedings until the cell was capped. Control colonies roughly doubled their feeding effort with each LDD except the last. The mean cumulative feeding visits (Fig. 3e; upper ends of coloured boxes, y-axis) were 4.4 ± 0.3, 11.5 ± 0.9, 22.7 ± 1.4, 45.8 ± 2.1, 93.2 ± 3.5 and 112.5 ± 2.4 on LDDs 1 to 6, respectively. As shown in Fig. 3j, feeding visit duration was strongly correlated with feeding visits. Controls fed for a mean of 8.9 ± 0.6, 13.8 ± 1.5, 24.7 ± 2.1, 47.8 ± 2.3, 71.4 ± 2.4 and 26.0 ± 6.5 minutes on LDDs 1 to 6, respectively. Cumulative feeding duration was 8.9 ± 0.6, 22.7 ± 2.1, 47.4 ± 4.1, 95.2 ± 5.4 and 166.6 ± 6.4 minutes on LDDs 1 to 5, respectively, and the total feeding duration was 192.6 ± 4.0 minutes at cell capping on LDD 6. These values include the varying inspection times preceding food provision. In the mean of treated colonies cumulative feeding visits were lower than the control starting with the second development day (Fig. 3e). This tendency was increasing with every LDD but was levelled throughout LDD 6. Concomitant results were present for feeding duration (Fig. 3j). Treated colonies showed reoccurring patterns between May and June (Figs. 3a–c and f–h) but not in July (Fig. 3d,i). Up to LDD 5, 200 ppb thiacloprid treatment showed less daily and cumulative feeding visits and durations than the control. This was also the case for 10 ppb clothianidin in May and June 2016, and for 1 ppb clothianidin treated colonies up to LDD 4 in all experiments except in May 2018. On LDD 6 however, more daily feedings were counted in the treatments in 10 of 12 cases, hereby decreasing the differences to the control in total feedings until capping. In July 2016 the thiacloprid treatment was at level with the control and exceeded its feeding visits (Fig. 3d) and durations (Fig. 3i) on LDDs 4 and 5. In this experiment, treatment with 1 ppb clothianidin reduced nursing parameters more than 10 ppb clothianidin. Compared to the other experiments, the nursing parameters were highest on LDDs 1–5 and lowest on LDD 6 in all colonies of July. For all parameters in Fig. 3, out of 195 post-hoc comparisons to the control, 69 remained significant and 13 were above the threshold after *p*-value adjustment (see Supplementary Table S1).

Figure 4b,c illustrate mean cumulative feeding visits and durations, respectively, as a function of neonicotinoid treatment five days after the first feeding (same as upper ends of LDD 5 boxes in Fig. 3). The one-way ANOVA showed that the number of feeding visits were significantly different for the treatments in May 2016 (*F*(3, 67) = 5.01, *p* = 0.003), May 2018 (*F*(3, 110) = 7.18, *p* < 0.001), June 2016 (*F*(3, 25) = 6.72, *p* = 0.002), and July (*F*(3, 16) = 4.22, *p* = 0.022) but not the mean (*F*(3, 12) = 1.43, *p* = 0.282). While in July 2016 no statistical differences were present in pairwise comparisons, 10 ppb clothianidin significantly reduced feeding visits in May 2016 (t = 2.813, *p* = 0.024, n = 17) and June 2016 (t = 3.114, *p* = 0.018, n = 14), and 200 ppb thiacloprid in May 2016 (t = 2.820, *p* = 0.023, n = 21) and May 2018 (t = 4.002, *p* < 0.001, n = 29). Similar significances were present for feeding duration in May 2016 (*F*(3, 67) = 3.98, *p* = 0.011), May 2018 (*F*(3, 110) = 7.64, *p* < 0.001), June 2016 (*F*(3, 25) = 5.06, *p* = 0.007), and July (*F*(3, 16) = 4.68, *p* = 0.016) but not the mean (*F*(3, 12) = 1.15, *p* = 0.370). Again, no statistical differences were present in pairwise comparisons in July. 10 ppb clothianidin reduced feeding duration in June 2016 (t = 3.006, *p* = 0.023, n = 14) but not in May 2016 (t = 2.126, *p* = 0.082, n = 17), and 200 ppb thiacloprid in May 2016 (t = 2.586, *p* = 0.041, n = 21) and May 2018 (t = 3.932, *p* < 0.001, n = 29). In June 2016 only 6 larvae in the control and 4 larvae in the thiacloprid treatment were available for analysis. Corresponding to the small number of larvae, tested differences were not significant.

### Development time

In 2016 and 2018 we measured the feeding timespan as an indicator for development time, since timestamps of the first and last feedings were available in all analysed cells. To calculate the real development times, oviposition, larval hatch and cell capping timestamps were required. Since oviposition required between 10 to 20 seconds, its detection on our images was principally possible, and easily determined by a sudden appearance of the egg in the cell. However, this was not the case in all cells that we used for analysis. Sometimes the egg and subsequent larval hatch were hidden from sight due to the viewing angle, and some recordings started with eggs already present in the cells. In numerous cases, eggs were pushed down during the first three days of development and disappeared from the field of view after a worker bee entered and appeared motionless the cell (suspected as heating events; in some cases larval hatch occurred during this period). Therefore, we measured the feeding timespan.

One-way ANOVA displayed a statistically significant difference in feeding timespans for the treatments (Fig. 4a) in May 2016 (*F*(3, 67) = 6.18, *p* < 0.001), May 2018 (*F*(3, 110) = 4.82, *p* = 0.003) and June 2016 (*F*(3, 25) = 6.04, *p* = 0.003). We observed that means were negatively correlated to feeding visits and duration (Fig. 4b,c, respectively) and positively correlated with nursing activity on LDD 6 (Fig. 3). Thiacloprid treatment increased the feeding timespan in May 2016 from 5.33 ± 0.09 to 5.74 ± 0.07 days (mean ± SEM, t = −3.656, *p* = 0.002, n = 21), and in May 2018 from 5.31 ± 0.05 to 5.54 ± 0.07 days (t = −2.571, *p* = 0.039, n = 29). 10 ppb clothianidin increased the feeding timespan in June 2016 from 5.45 ± 0.14 to 5.89 ± 0.07 days (t = −3.053, *p* = 0.021, n = 14). The remaining 13 of the 16 post-hoc comparisons were not significant. We found no significant difference between the means of all experiments (Fig. 4a, Mean) (*F*(3, 12) = 0.72, *p* = 0.560), possibly due to the low amount of tested colonies.

We examined if differences in feeding timespans depend on the exposition time to the substances via the feeding solution (Fig. 5). Feeding timespan was statistically different for the timespan between experiment initiation and the first feeding of the larvae in the control (*F*(1, 55) = 29.00, *p* < 0.001), 1 ppb clothianidin (*F*(1, 50) = 4.52, *p* = 0.038), 10 ppb clothianidin (*F*(1, 64) = 10.51, *p* = 0.002) and thiacloprid treatment (*F*(1, 57) = 30.76, *p* < 0.001). For example, control larvae that were fed for the first time after 7 days into the experiment received feedings throughout 5.7 days until the cell was capped. 14 days after the start of the experiment, larvae were fed for only 5 days. We found that homogeneity of regression slopes was preserved between treatments (one-way analysis of covariance, ANCOVA; *F*(3, 226) = 1.66, *p* = 0.177) suggesting that there was no time cumulative effect of the neonicotinoids in the two weeks of feeding larvae in the first cell layer (between 7 and 21 days). Therefore, the seen increase in feeding timespans in the treated colonies (Fig. 4a) were due to effects present shortly after experiment initiation and did not build up remarkably over the time course of the experiments.

As the prolonged feeding timespans in treated colonies (Fig. 4a) indicated a delay in ontogenesis, we further investigated colonies that were fed high concentrations of clothianidin (100 ppb) and thiacloprid (8800 ppb) using ontogenesis timestamps (oviposition, larval hatch, cell capping, emerge) in 2017. Unfortunately, the number of successful experiments was limited to one due to the two following reasons. Firstly, observing the complete ontogenesis from oviposition to hatch of the imago took additional two weeks experiment time. Secondly, colony development was obviously retarded in colonies with high neonicotinoid dosages, thus delaying recordings of the first layer. For example, on June 9^th^ 2017, 17 days after start of the experiment, the colony treated with 100 ppb clothianidin had only three capped cells in the visible cell layer, whereas the control had 75. We terminated the experiment on July 14^th^ after which we were able to determine necessary ontogenesis stages in 8, 13 and 10 cells of control, 100 ppb clothianidin and 8800 ppb thiacloprid treatments, respectively. We had only 8 control cells which were developing in the same period of time as the delayed treated colonies – most other brood cells in the control colony were already capped. As shown in Fig. 6, we classified four observable worker ontogenesis stages. The first, from oviposition to larval hatch (egg); the second, from larval hatch to completed cell capping (uncapped larva); the third, from cell capping to the beginning of metamorphosis (capped larva); and the fourth, from metamorphosis to the hatch of the imago (pupa). Complete ontogenesis from oviposition to hatch of the imago required 20.50 ± 0.07 days (mean ± SEM, n = 8) in the control colony. One-way ANOVA showed that development times of egg (*F*(2, 28) = 19.44, *p* < 0.001), uncapped larva (*F*(2, 29) = 27.89, *p* < 0.001) and pupa (*F*(2, 29) = 36.90, *p* < 0.001) stages and of complete ontogenesis (*F*(2, 29) = 14.35, *p* < 0.001) were significantly different for the treatments. In pairwise comparison to the control, statistically significant differences were present in the 100 ppb clothianidin treatment for egg (t = −8.396, p < 0.001, n = 13), uncapped larva (t = −6.518, p < 0.001, n = 14) and pupa (t = −4.755, p < 0.001, n = 14) stages and complete ontogenesis (t = −4.217, p < 0.001, n = 14), and likewise in the 8800 ppb thiacloprid treatment (t = −3.984, p = 0.002; t = −4.925, p < 0.001; t = 2.915, p = 0.010; t = −3.390, p = 0.004, respectively, n = 10). All of the 8 post-hoc comparisons were significant, while the ANOVA of the capped larva stage showed no statistical differences. Development times were increased in all cases, except for pupal development in the thiacloprid colony, which was surprisingly shortened. The shown delay in embryo development suggests a mechanism independent from feeding behaviour of nurses. These results support our previous observations of retarded brood development in our low concentration experiments. However, one should keep in mind that data is from only one colony each and that larval development time could have been influenced by the obvious differences in brood area development (3 to 75 capped cells after 17 days as described above; cf. Fig. 5).

Unrepeated weighting experiments in the low concentration experiment in May 2018 also showed delayed development. Upon correlating weight and development duration in 22 uncapped cells per treatment we determined that larvae in the 200 ppb thiacloprid treated hive required 10.8 hours longer than the control to gain 150 mg of weight (regression in control: y = 7E^−7^ × ^9.2365^, R^2^ = 0.89; thiacloprid: y = 2E^−5^ × ^7.4259^, R^2^ = 0.92). Larvae in the 10 ppb clothianidin treatment required 5.4 hours longer (y = 2E^−5^ × ^7.5218^, R^2^ = 0.93). In general, larvae weight between 137.6 and 158.3 mg at cell capping^42^.

### Food uptake

We tested whether the neonicotinoid-spiked sugar solution was taken up by colonies by regular weighting of the feeder. Control hives collected between 2,101 and 2,766 g (mean ± SEM: 2,452 ± 184 g, n = 4) of sugar solution during experiments. Colonies treated with 1 ppb clothianidin, 10 ppb clothianidin and 200 ppb thiacloprid collected 2,299 ± 229 g, 2,320 ± 245 g, and 2,094 ± 251 g, respectively (four colonies per treatment). One-way ANOVA showed no significant differences in food uptake between treatments (*F*(3,12) = 0.42, *p* = 0.743).

## Discussion

Our novel video recording and analysing methods have proven successful to process a comprehensive amount of honey bee brood care behaviour continuously over several weeks. For the first time, we were able to demonstrate significant differences of nursing behaviour and larval development time from within a functioning hive that was chronically fed with neonicotinoids. Notably, in May (Fig. 3a,b and f,g) statistical differences in nursing amount and duration were present already during the initial days of development. Furthermore, treatment with 200 ppb of the partial nicotinergic agonist thiacloprid did not induce opposing results compared to 10 ppb of the more effective agonist clothianidin. In June (Fig. 3c,h) only few cells were analysable in the control and the thiacloprid treatments and statistical differences in daily and cumulative nursing parameters were missing. We observed high nursing activity in the initial LDDs of July (Fig. 3d,i) and found no significant differences between the means of all experiments (Fig. 3e,j; Mean). In conclusion, we can neither clearly confirm nor refute a neonicotinoid effect on nursing behaviour and larval development throughout all experiments, although we observed various indications of such effects. This includes experiments using high neonicotinoid dosages in which brood area development was obviously retarded and developmental stages were delayed (Fig. 6). A further factor influencing development time was the duration between experiment initiation and the first feeding of the larva. We chose all larvae for analysis without bias towards this factor as shown in the distributions in Fig. 5. The effect was treatment independent and could be related to the amount of capped brood within the colony. We conclude that there was no accumulating effect by the neonicotinoids from 7 to 20 days that further retarded larval development. It should be further investigated if the results of July were due to seasonal circumstances and if hives rear brood differently, as shown in the relatively high daily feeding visits and feeding duration compared to the other experiments conducted in May and June. We want to emphasize at this point that our hive architecture was optimized for observation and video recordings, with a relatively small amount of colony members. Although the validity of the observed effects within managed full colonies remains to be proven, we, nevertheless, were able to quantify social behaviour in the brood area within a hive that was stable throughout the whole study period of up to six weeks. Therefore, we assume that our conclusions can be assigned to full-sized colonies with the given caution based on the different hive dimensions.

To date, investigations on neonicotinoid effects have focused on neuronal impairments (see introduction for references, review^22,23^) because acetylcholine is the most abundant neurotransmitter in the insect brain^43^ and is used in various brain regions of the honey bee, such as the antennal lobes and mushroom body^44^. Therefore, alterations in behaviour are expected. Publications noting high non-neuronal acetylcholine concentration in brood food^30,32–34^ suggest neonicotinoid impairments of development as a further possibility. The cholinergic system is suggested to be important in insect development^45^ but the non-neuronal cholinergic system of insects is largely understudied. Various delaying effects by neonicotinoids and other insecticides targeting the cholinergic system, such as dimethoate, monocrotophos, methylparathion or quinalphos, have been demonstrated elsewhere (for review see^24^). Currently, the targets of non-neuronal acetylcholine, how it affects honey bee larvae and its modes of action remain to be discovered. However, it should be noted that other nest-mates and the queen are also fed by nurses^46,47^, thus broadening the range of possible targets of non-neuronal acetylcholine within the hive.

In this study we have shown indications of delayed development using various neonicotinoid concentrations within our hives, as shown in Figs. 4a and 6. However, because larval development and nursing behaviour are closely linked to each other, we do not know whether the tested neonicotinoids primarily affected nurses, larvae or both at the same time and had no possibility to disentangle these possibilities in the shown experiments of this study. Furthermore, we do not know whether alterations in A) behaviour, B) development, or both are the underlying causes.

Regarding A), behavioural alterations, the reduced number of feeding visits and durations within the treatments, presented in Fig. 3, could have been the reason for the prolonged feeding timespan, and thus, brood development observed. In this case, neonicotinoids would have had a negative effect on the nervous systems of nurses to such a significant degree that they affected feeding behaviour and/or induced incorrect decisions. It is expected that nurses adjusted food composition^40,48–50^ and nursing activity^51,52^ according to the condition and developmental stage of the larvae. Previous studies have shown a correlation between nursing duration and the development time of larvae^42,53,54^. On the other hand, queen larvae that are fed more often than worker larvae have a lower mean weight between hours 6 and 90 of development^55^. This suggests that food amount between castes is not the exclusive factor for affecting growth, but that growth rate may instead be linked to food composition. Although we were unable to measure the amount of food provided during each visit, there were no differences observed in feeding durations over the first three days (Fig. 2). Assuming that food amount is positively correlated with the time spent in a cell during feeding, we presume an effect on development duration based on alterations in the amount of food provided was not being present. The mean total of 112.5 ± 2.4 nursing visits in the control colonies is below observations of previous studies in this field^49,56^. However, we postulate that our values are the most precise thus far due to a greater sample size with automated analyses.

Other brood rearing relevant behavioural impairments, such as irregular inspections or the inability of temperature preservation, could also have induced the observed delay in development and further analysis of these behaviours using our video data is intended. Temperature has been shown to have an influence on development time^57^. The surrounding room temperature was 26.9 ± 1.4 °C, 26.9 ± 1.7 °C, 27.6 ± 1.4 °C and 29.5 ± 1.3 °C in May 2016, June 2016, July 2016 and May 2018, respectively (mean ± SD, start to end of the experiments, 30 minutes reading interval). We also derived the standard deviations of temperature measurements across each individual day. These standard deviations ranged from 1.5 (May 2018) to 4.3 (May 2016), with a mean standard deviation of 3.0 for all experiments in 2016 and 3.1 for May 2018. Hives were approximately 4 °C above room temperature (within-hive sensor were placed on the floor in the brood area, level with the entrance). We were unable to accurately determine whether primarily motionless bees displayed heating events, although these events were not observed in a hive with over-heated surroundings (this hive was at a different location). In an unpublished study, we delayed the development of larvae reared under constant temperature *in vitro* using high concentrations of imidacloprid in our lab; therefore, temperature is unlikely to be the only reason for retarded larval development in our experiments. Alternative possibilities include chemical miscommunication, for example by impaired perception of nurses or by impaired emittance of larval pheromones. Since the mean of the experiments had similar values in cumulative amount and duration on LDD 6 (Fig. 3) the nurses presumably perceived the state of the larva at the time point of capping correctly.

Regarding B), nurses could have adapted their behaviour because of impairments in larval development, if sensory perception was well functioning. Since the direct transmission of neonicotinoids from nurse to larva seems to be very low^58^, a further possibility is that development within the nurses was affected, and that food secreting organs were impaired. Neonicotinoids have been shown to influence the size^28^ and activity^30^ of food-producing glands (for review see^24^) and underlying endocrine controls within age polyethism^59^. For example, the development of hypopharyngeal glands is retarded in caged bees using 2 and 3 ppb imidacloprid in a sugar solution and pollen pastry, respectively^28^. Unpublished results from our lab using colonies systematically treated with high concentrations of clothianidin (100 ppb) and thiacloprid (8800 ppb) suggest similar effects. Hypopharyngeal gland volume of seven-day-old workers was significantly reduced, glands appearing white and undeveloped as of newly emerged bees. Gland impairments could affect food quality, for example, affecting non-neuronal acetylcholine concentrations found in the jelly^30,32–34^. However, retarded development may be independent of gland secretions and feeding behaviour of nurses, since eggs in colonies treated with high neonicotinoid concentrations showed a developmental delay (Fig. 6). Yet, queens previously received feedings by the workers as well. Because development is mainly mediated by hormones, we should also envisage an impairment of endocrine organs within larvae and/or nurses, as a couple of studies have suggested up to date^60,61^. This should be investigated in further analyses.

We anticipate that our versatile method can be used for a variety of behavioural monitoring studies that require precise time determination under various stressor influences. Apart from general effects from other beekeeping chemicals, this includes bees’ reactions to parasites such as *Varroa destructor* under various conditions, and, for example, after the extraction and insertion of food or chosen substances into individual cells.

## Materials and Methods

### Animals and colonies

**Figure 7:**
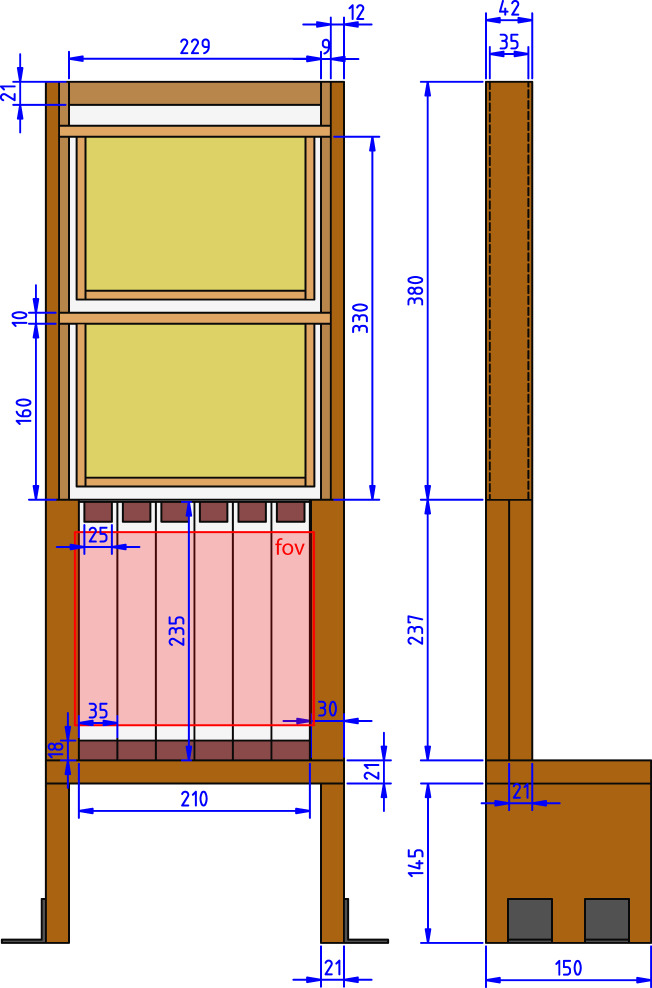
Technical drawing of the observation hive. The recorded brood area (fov = field of view) contained six glass stripes (light grey / red overlay) to mount combs (not shown), which were pressed against the glass using metal bars (not shown) across top and bottom wood blocks (deep red). The upper food super contained two MiniPlus-sized frames (yellow) and was separated from the brood area by a queen grid above the glass stripes. The food super was visible though embedded acrylic glass. The hive was fixed at the bottom using angle connectors (dark grey).

We used small observation hives (outer dimensions H × W × D: 617 × 271 × 42 mm; Fig. 7) that were each filled with 300 g *Apis mellifera carnica* (approximately 3,000 individuals) taken from bee hives of the Institut für Bienenkunde in Oberursel as well as one queen. Sister queens were taken from our on-site queen breeding programme. Hives were divided by a queen grid into an upper food super (inner dimensions H × W × D: 359 × 229 × 35 mm) and a lower brood area (inner dimensions H × W × D: 235 × 211 × 35 mm) by preventing the queen from entering the food super. At the start of the experiments it contained two wooden MiniPlus-sized frames with mounted comb foundations, while visual inspection was granted by acrylic glass. The brood area had six “comb stripes” (35 mm stripes of an empty well-constructed Zander frame comb) that were turned 90° to allow video observation of the truncated cells through a 4 mm layer of anti-reflective glass (Energy Vision 1, Hecker, Dortmund). This enabled the continuous recording of nursing behaviour and developing worker bees from oviposition until adult eclosion within individual cells (Fig. 1b). We further developed this concept of looking sideways into the cells from a previous publication^56^. All wax that was set into the hives was previously treated with a toxin against wax moths (B401, Vita Europe Ltd, Basingstoke) to prevent their development during the experiments. One comb stripe had a maximum of 40 vertical cells per side. Bees had six or seven horizontal cells for brood rearing, alternating due to offset comb structure. Therefore, a comb stripe had a maximum of 520 cells and a total of 3120 cells within the brood area (six comb stripes). As shown in Fig. 1b, the camera observed approximately 70 vertical cells in each comb stripe and a total of 420 in the field of view. Therefore we observed approximately 13.5% of all available cells in the brood area. A comb stripe was laterally confined by two glasses and vertically by two wood blocks. Serving as spacer, the wood blocks allowed firm attachment of the glass to the comb stripe. To create passages to the food super the upper blocks were narrower (H × W × D: 18 × 25 × 35 mm) than the bottom one (H × W × D: 18 × 35 × 35 mm).

### Treatments

We simultaneously recorded four hives with various treatments: control, 1 ppb clothianidin, 10 ppb clothianidin and 200 ppb thiacloprid (Sigma-Aldrich Chemie GmbH, Munich). The hives were positioned in a dark and temperate (~27 °C) room whose temperature was continuously monitored. With a feeder on top of the food super, we applied control sugar solution (ApiInvert, Südzucker AG, Germany + 0.0002% acetone) or in acetone dissolved neonicotinoids in sugar solution. Since feeding started with experiment initiation, all bees were potentially exposed to the treatments from the first experiment day. The weight of the feeder was measured before refilling. Hives were connected to the outside using a tunnel in the brood area. After a maximum of three weeks there were sufficient capped cells for further analysis. We then stopped the recordings and fully disassembled the hives, sweeping all colony members into an artificial swarm box. Queens were not in the swarm. We extensively cleaned the hives with a brush and warm water to remove remaining food. Feeders were cleaned with warm water as well. We then used a new set of six well-constructed comb stipes and two MiniPlus-sized frames with mounted comb foundations for the next repetition. We refilled the colonies with new bees as described above on the following day to the end of a three week experiment. We restarted the experiments as long as the brood season (February – October in Germany, coordinates: N50°13′4.296″ O 8°32′54.024″) permitted or until too little cells at the glass were occupied by developing larvae with decreasing offspring (typically between August and September). Sister queens were available from May. Due to this effectively to three months limited experiment time per year we did not keep track of adult emergence, since this would have required another 12 days. However, we followed the full worker ontogenesis using high neonicotinoid concentrations (100 ppb clothianidin and 8800 ppb thiacloprid) in 2017 on cost of the number of possible repetitions.

### Video registration and data analyses

We evaluated a total of 26,483 feeding visits in 234 brood cells of 16 hives (number of cells in control: 57; 1 ppb clothianidin: 52; 10 ppb clothianidin: 66; 200 ppb thiacloprid: 59), recorded in four repetitions and two experimental years. In 2016 experiments were from May 17^th^ to June 6^th^ (May 2016), from June 7^th^ to June 27^th^ (June 2016) and June 28^th^ to July 18^th^ (July 2016). In 2018 one experiment from May 14^th^ to June 1^st^ (May 2018) was available. A total of 60 cells were analysed using manual classification (6,539 feeding events) and 174 were analysed with a convolutional neural network algorithm (19,944 feeding events). Nursing behaviour within honey bee colonies and its analysis within our experiment arrangement was evaluated by the following assumptions: Firstly, nursing always takes place within the comb cells; secondly, all bees within the colony are of approximately the same size and appearance (i.e. patterns and colours); and thirdly, workers require a certain amount of time within the cell for nursing. Given these strong constraints, we had strong expectations regarding which video pixels were going to change over which timeframe within the fixed comb structure upon a nurse entering a cell to feed. Therefore, we were able to compress our video data from three dimensions (width, height and time) into two (width and time) without losing important information, and were further able to record at one frame per second. For 2D compression we followed a systems engineering methodology for the design of the video analysis system^62^.

Key factors that influence the design of a vision system include sensor choices, application contexts, as well as tasks and other requirements such as hardware constraints and costs (among others; Table 1). Some important parameters for the contextual setup of data collection for hive observations include (from and Fig. 1a): cd – camera distance from brood area, dl – dome lighting, h_1_ – brood area height and sc – height of sub-construction. The recording arrangement was constructed by setting these parameters as follows: To increase the likelihood of developing larvae within the visible cell layer, we balanced the arrangement to record a large area of cells and preserve sufficient video details for later analysis (for video resolution see Supplementary Video S2). Brood area height was adapted from the Zander-frame height (205 mm) plus additional 30 mm space to place wooden spacers at the top and bottom for comb fixation (see red rectangles in Fig. 7). Brood area width was set to create the most approximate square by multiplying the 35 mm glass-stripe width (space to include combs and inter-comb spacing for bees to pass) by 6, resulting in 210 mm. For optimal illumination, we constructed a dome-light size that encircled our observation area and set the height of the sub-construction to align the centres of the observation area and the dome lighting.

**Table 1:** Tasks and application requirements to record nursing within a hive.

Tasks	Application Requirements/Constraints	Design Choice
Long term recording	Limited hard disk storage space	Sample video at 1 fps/JPEG real-time compression
Honey bee detection	Good contrast of background foreground required	Use of bright empty honey combs & red light
Sufficient video resolution & number of observable cells	Analysis requires acceptable image quality	Balanced camera distance/5.3 MP camera/4.8 µm pixel spacing
Record without shadows through glass	Reflections occur on glass	Usage of dome lighting
Keep hive naturally dark	Bees might get disturbed by visible light	Usage of 660 nm red light/monochrome cameras
Observe nursing within cells	Feedings occur within cells; constrained cell-entering possibility	Compress 2D + time data to 1D + time data by projections along a line interval orthogonal to the cell entry direction.
Analyse larval development time	Development time is linked to temperature	Control surrounding room temperature

**Figure 8:**
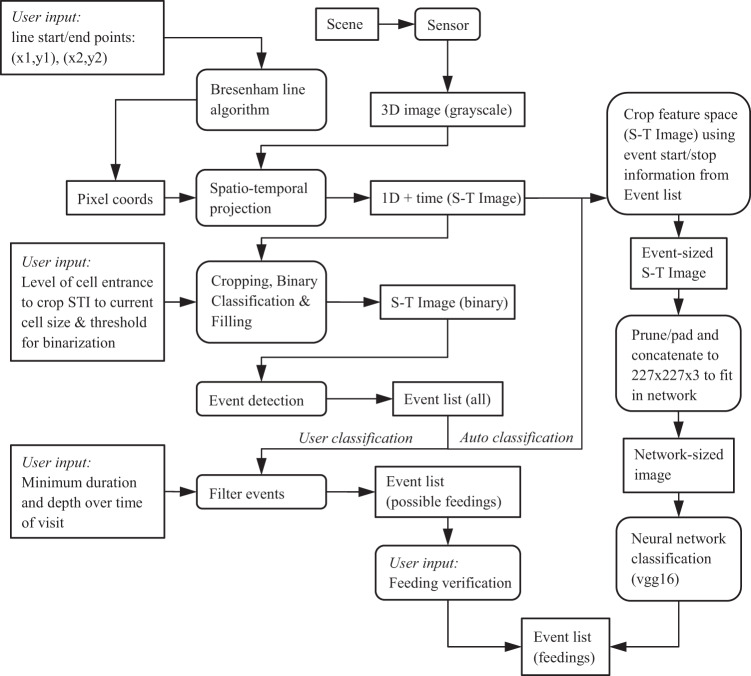
Object-process diagram for our application: Boxes with rounded corners represent transformations, while regular boxes represent data objects. We used a manual classification (user classification) and used the resulting and further classified image data to develop an automated STI classification using a neural network algorithm (for more information on the Object-process Methodology, see^65^).

We recorded the brood area using a 5.3 MP monochrome USB3 camera (PL-D725MU-T, PixeLINK, Ottawa), a C-mount lens with 12.5 mm focal length (LM12HC, Kowa Optical Products Co., Ltd., Tokyo) and a red light-emitting dome-light beyond the range of honey bee colour vision (λ_peak_ = 660 nm). One comb stipe was recorded with 2048 × 420 pixels (H × W). The 1″ camera sensor fulfilled photon detection requirements under monochrome lighting conditions. Recording at one frame per second was reasonable to sufficiently observe nursing behaviours and minimized the amount of required hard-disk space, thus allowing continuous recording of the four experiment arrangements simultaneously throughout the season. However, we acquired high temporal resolution cameras to ensure the flexibility of experimental arrangements. StreamPix (version 6.3.0.155 and 7.4, NorPix Inc., Montreal) was used as recording software. For analysis, we used a manual or automated classification process shown in the block diagram of Fig. 8. While one could process video frames at the original resolution, the application context, the analysis goals and computational requirements motivated us to perform projections of videos (2D + time) into images (1D + time). The space-time image (STI) constructed is presented in Fig. 1d. For STI generation the brightness information of the cells centre pixels from the bottom to the entrance (using Bresenham’s line algorithm; Fig. 1c) was concatenated over time. Entering bees appeared as dark structural change on the STI (from now: ‘events’). STI representation has been used in a number of real-world video monitoring tasks (e.g^63,64^). In this work, we simply concatenated the pixel values selected at the observation lines. The temporal properties of the filtered signal retain relevant information regarding the dynamics in the video due to illumination variation, shadows, reflections, occlusions and background motion. However, the STI representation clearly shows features that facilitate the classification of these effects based on visual patterns.

Because the three-week recordings had over one million frames, STIs were divided into groups of 65,499 frames to enable common display possibilities. In a programmed graphical user interface (MATLAB 2014a – 2018a by MathWorks, Natick) running on Windows 7 we stored events if brightness was below a threshold (user adjusted for best detection, bees appeared darker than the background wax which was variable in brightness). To narrow down possible feeding events during manual classification, events were filtered by duration (10–300 seconds) and depth over time (bees use at least 65% of available space in a cell over a user-defined amount of time). These filters were applied prior to consecutive verification by hand. In case of doubt, comparisons were made with the corresponding video material.

We observed that events on the STIs had a similar pattern appearance according to the behaviour performed by bees. Therefore, feedings could usually be characterised quickly without consideration of the video footage during manual classification. We used this circumstance to train a convolutional neural network (Vgg16) with 63,411 truncated event images we had classified for this purpose. We trained the algorithm to discriminate between “feeding” (19,472 manually classified events used for training; including 6,539 of the 2016 experiments), “heating” (10,691 events), “building” (28,149 events) and “unknown” (5,099 events) by analysing patterns and structures within the event STIs (for behaviour characterisation see results). The images were normalised in brightness and contrast and then cropped or padded black and concatenated to fit a specific size (227 × 227 × 3 pixels; height, width, colour channels) in the neural network input layer. Although this shortened some events to 227 seconds (pixels), the important information was sufficiently preserved. Behavioural changes within events were extremely rare. For network training all image labels were split into two random batches, one for algorithm improvement and the other one for testing accuracy. Although our two-class neural network (feeding/unknown) was able to detect feedings with 99.4% accuracy, we chose an analysis with four classes (95.9% accuracy) in order to also analyse the effects on other behaviours in the future. During neural network classification we bypassed the maximum visit length filter (set from 300 to 10,000 seconds) and renounced depth over time filtering to include long lasting heating events in the list (“Event list (all)” in Fig. 8). Afterwards, we excluded incorrectly classified feeding events above 300 seconds from the final event list. Code has been made available at: https://github.com/paulsiefert/honey-bee-behaviour.

### Statistics

Statistics were calculated using IBM SPSS Statistics, version 23.0.0.2, with α = 0.05. We applied decimal logarithm transformations on dependent variables “Feeding visits”, “Cumulative feeding visits”, “Feeding duration” and “Cumulative feeding duration” to normalize positive skewness. Dependent variables “Feeding visit duration”, “Feeding timespan” and “Development time” were not transformed. Because we expected altering nursing parameters throughout larval development data were split into six larval development days (LDDs). We ran statistics on each experiment individually and the means of all experiments (Mean) to allow a differentiated assessment of the statistical results by each reader. For each dependent variable we performed a one-way ANOVA. If significant, we used the Student’s *t*-test to compare each of the three neonicotinoid treatments to the control. If the *t*-test significance was below 0.05 we adjusted significance levels using Bonferroni-Holm type I error-correction. The lowest significant *p*-value was multiplied by three, the second lowest by two and the third by one. We did not compare between neonicotinoid treatments, therefore, the number of tests used for significance level adjustment was three. The same tests and corrections were used for development time in 100 ppb clothianidin and 8800 ppb thiacloprid treated colonies in 2017, here the number of tests used for significance level adjustment was two. Statistical results in the text are given in the following format: F(df_numerator_, df_denominator_) = F-value, *p-*value; df = degrees of freedom.

## Supplementary information

Supplementary information.

Supplementary video S1.

Supplementary video S2.

Supplementary video S3.

## Data Availability

The datasets generated during and/or analysed during the current study are available from the corresponding author on reasonable request.
