# Supplementary material for: Chronic within-hive video recordings detect altered nursing behaviour and retarded larval development of neonicotinoid treated honey bees
Source: Sci Rep. 2020 May 26;10:8727. doi: 10.1038/s41598-020-65425-y (PMC7251098; doi:10.1038/s41598-020-65425-y)
Supplement: Supplementary file 1 — Supplementary information. [file 41598_2020_65425_MOESM1_ESM.pdf]

Supplementary material

Chronic within-hive video registrations detect altered nursing behaviour and retarded larval development of neonicotinoid treated honey bees

Paul Siefert[1*], Rudra Hota[2], Visvanathan Ramesh[2] and Bernd Grünewald[1]

[1]Institut für Bienenkunde
Polytechnische Gesellschaft Frankfurt am Main
Goethe-Universität, Frankfurt am Main, Germany

[2]Center for Cognition and Computation
Institut für Informatik
Goethe-Universität, Frankfurt am Main, Germany

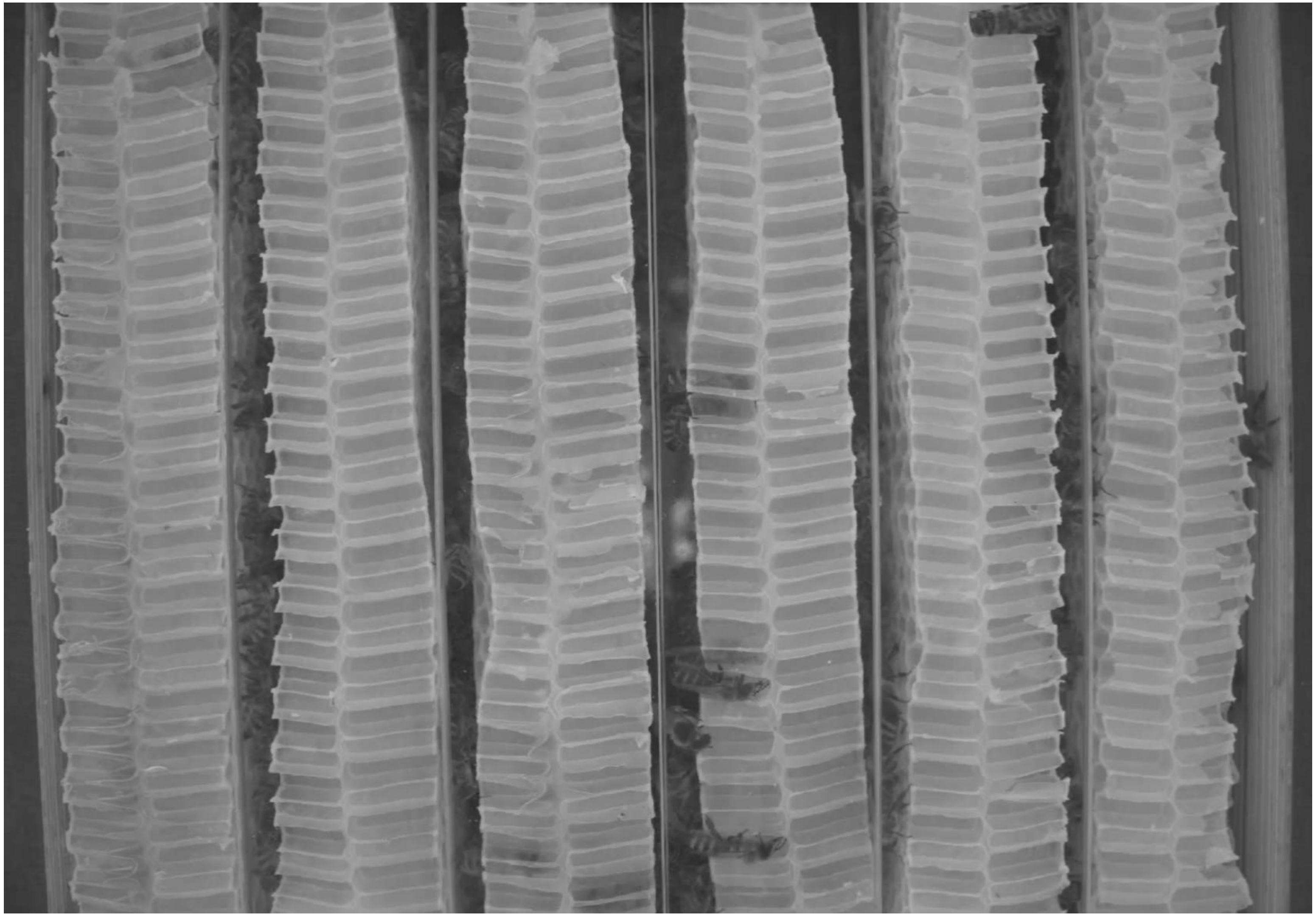

Supplementary Video S1. Time-lapse of initial building activity after setup of a hive.

This recording of a full-sized camera view in a pre-experiment in 2015 started right after setup of the hive. The wax of the cells adjacent to the glass (first cell layer) of the provided well-constructed combs was remodelled to fixate the combs to the glass. Therefore, the cells of the first cell layer initially appear to shrink in size and are extended again afterwards. In later experiments, the full-sized image was divided into six images as shown in Supplementary Video S2 to increase performance of the space-time image generation script. Furthermore, this initial building phase was not recorded to save hard-disc space since no feeding behaviour was present. Over the course of the video, adjustments were being made to the camera hardware to optimise image brightness, contrast and hard-disk requirements (capturing frequency). This material is protected by copyright. All rights reserved.

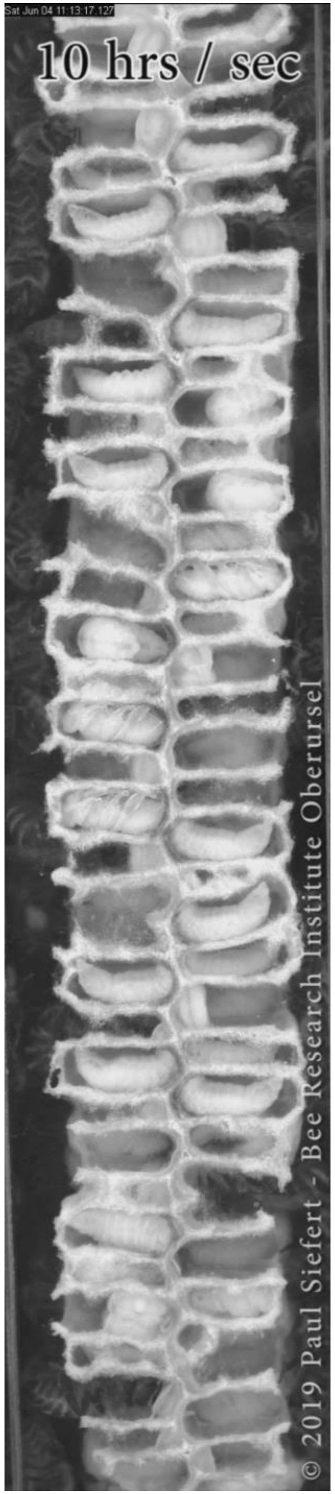

Supplementary Video S2. Comb stipe in a control colony, demonstrating the development in the breeding area within the observation hives.

This video footage was used for analysis. However, quality has been reduced to enable online publication. The colony was setup on May 17th 2016. Recording started on May 22nd and ended on June 5th, as displayed in the upper left corner. We recorded with 1 frame per second throughout the experiment, as shown in the initial 14 seconds of the video (running speed displayed at the top). Afterwards, the video is in time-lapse to demonstrate colony development. Eggs were visible shortly after colony setup and appeared descending only when workers pushed them during a deep visit in the cell, or when the larva hatched. As seen in numerous cells, young larvae of the initial eggs were cannibalized (though initially fed) until development in the invisible inner cells progressed to a certain degree (almost all inner cells were capped when colonies were disassembled at the end of the experiment). After observable development in the second layer of cells, nurses started to rear brood in the first layer shortly after, displaying that inner cells were prioritised.

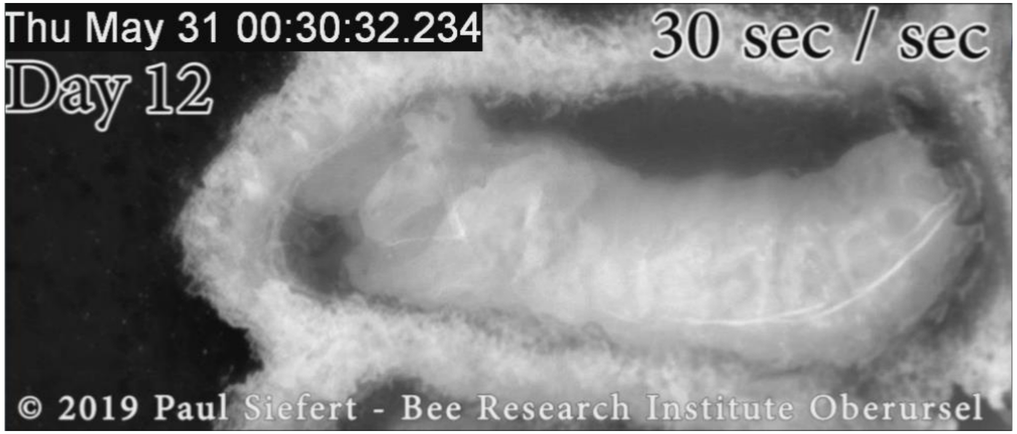

Supplementary Video S3. Complete worker ontogenesis within a truncated cell. This video was recorded from May – June 2018 for illustrational purposes in a setup specially designed for macro recordings. On the top right is the playback speed. On the top left is the date and below it the day of worker ontogenesis. Initially, the queen inspects the cell with frequent antennal movements, which are also characteristic for inspections by workers. Hereafter, the queen places an egg at the bottom of the cell. The video now focuses a different cell in which oviposition was not recorded. We approximated the larval hatch after 72 hours of development in this cell. Workers showed release of a clear substance on the glass that was taken up shortly afterwards. We assume cooling purposes since the colony stood in a very well-heated location and this behaviour was not observed in experiment colonies. On the end of day eight, after five days of larval development, the cell is capped and cocoon spinning starts for about 36 hours. The prepupa state lasts approximately 48 hours showing some movement during the first of the two days. On day twelve pupal metamorphoses takes place: The larval skin is ripped open on the side of the individual from caput to abdomen, opening zipper-like within 20 minutes. Approximately an hour later, the pupa remains motionless until day 19. The last pupal ecdysis is observable at noon on June 7th and shortly after, the imago outstretches its third pair of legs to prepare for wing development. Within 10 minutes the wings are extended and the imago does some longitudinal movements for another 15 hours before perforating the cell capping during day 20 of development. The estimated total development time of about 19.5 days is below the mean duration described in literature, possibly due to setup circumstances.

Supplementary Table S1. Descriptive statistics of nursing parameters in all experiments and the mean of all experiments. Data is displayed in reversed larval development day (LDD) order to allow comparability with Figure 2 in the manuscript. Red cells represent significant ANOVA *p*-value. We used Student's *t*-test for post-hoc analysis. If significance level was below $\alpha = 0.05$ we adjusted *p*-values after Bonferroni-Holm for type I error-correction (number of tests used for adjustment was 3). Green cells represent significant differences to the control after *p*-value adjustment compared to the control; yellow cells show non-significant values after adjustment. Abbreviations: LDD = Larval development day; n = sample size; ANOVA = Univariate analysis of variance; SEM = standard error of mean; C = control; C1 = 1 ppb clothianidin; C10 = 10 ppb clothianidin; T200 = 200 ppb thiacloprid; Sig. = significance level of Student's *t*-test.

May 2016

		ANOVA		C		C1				C10				T200			
				n = 19		n = 14				n = 17				n = 21			
	LDD	F	*p*	Mean	SEM	Mean	SEM	t	Sig.	Mean	SEM	t	Sig.	Mean	SEM	t	Sig.
Feeding visits	6	6.62	0.0006	19.89	3.66	28.50	3.13	-2.38	0.0248	32.24	3.30	-2.71	0.0265	51.81	4.28	-3.62	0.0026
	5	4.82	0.0043	48.42	2.50	50.64	2.10	-0.80	0.4293	39.00	2.28	2.60	0.0414	40.76	3.11	2.08	0.0888
	4	5.13	0.0030	23.26	1.61	18.57	1.27	1.86	0.0728	19.65	1.53	1.49	0.1461	15.90	1.50	3.58	0.0029
	3	0.67	0.5748	9.68	0.73	8.50	0.65			8.35	0.65			8.67	0.71		
	2	5.97	0.0011	6.26	0.70	4.64	0.55	1.47	0.1509	3.06	0.25	4.06	0.0008	4.10	0.41	2.45	0.0383
	1	5.01	0.0034	4.16	0.43	3.79	0.43	0.37	0.7126	2.59	0.17	2.96	0.0195	2.67	0.30	2.76	0.0177
Cumulative feeding visits	6	4.58	0.0056	111.68	2.43	114.64	4.15	-0.53	0.5966	104.88	4.29	1.61	0.1165	123.90	4.01	-2.33	0.0763
	5	5.01	0.0034	91.79	4.85	86.14	3.59	0.54	0.5900	72.65	3.53	2.81	0.0162	72.10	4.91	2.82	0.0228
	4	5.12	0.0030	43.37	2.83	35.50	1.84	1.79	0.0840	33.65	1.80	2.52	0.0334	31.33	2.49	3.31	0.0062
	3	4.15	0.0093	20.11	1.50	16.93	1.25	1.30	0.2020	14.00	0.77	3.26	0.0076	15.43	1.13	2.42	0.0405
	2	7.87	0.0001	10.42	0.94	8.43	0.86	1.26	0.2163	5.65	0.32	4.48	0.0002	6.76	0.62	3.15	0.0063
	1	4.71	0.0048	4.16	0.43	3.79	0.43	0.27	0.7994	2.59	0.17	2.74	0.0326	2.67	0.30	2.66	0.0228
Feeding duration	6	5.92	0.0012	28.84	5.51	40.22	4.77	-2.27	0.0316	48.32	5.18	-2.89	0.0153	74.96	6.65	-3.36	0.0054
	5	4.32	0.0076	77.05	2.94	85.71	3.54	-1.88	0.0690	68.31	3.91	1.95	0.0597	71.30	4.03	1.38	0.1765
	4	5.81	0.0014	48.95	3.07	40.08	2.54	1.69	0.1011	46.13	3.05	0.44	0.6660	34.40	2.81	3.43	0.0044
	3	0.19	0.9059	20.44	1.54	18.65	1.24			18.98	1.41			19.64	2.14		
	2	4.73	0.0047	12.20	1.26	9.73	1.03	1.11	0.2770	6.61	0.57	3.65	0.0026	8.23	0.78	2.34	0.0496
	1	6.50	0.0006	8.20	0.91	7.43	0.82	0.31	0.7608	4.47	0.31	3.94	0.0014	5.61	0.64	2.44	0.0385
Cumulative feeding duration	6	2.38	0.0774	195.70	3.47	201.82	6.74			192.82	7.40			214.14	6.44		
	5	3.98	0.0114	166.86	7.21	161.60	5.56	0.29	0.7702	144.50	5.62	2.13	0.0817	139.18	7.87	2.59	0.0410
	4	4.07	0.0102	89.80	5.47	75.89	3.28	1.53	0.1373	76.18	3.75	1.61	0.1158	67.88	5.50	2.94	0.0167
	3	2.92	0.0403	40.85	2.95	35.81	2.35	0.99	0.3308	30.05	1.69	2.83	0.0232	33.48	3.01	1.91	0.0631
	2	8.96	0.0000	20.41	1.73	17.16	1.59	1.11	0.2764	11.07	0.58	4.90	0.0001	13.83	1.20	3.07	0.0078
	1	6.5	0.0006	8.20	0.91	7.43	0.82	0.31	0.7608	4.47	0.31	3.94	0.0014	5.61	0.64	2.44	0.0385

May 2018

		ANOVA		C		C1				C10				T200			
				n = 27		n = 28				n = 30				n = 29			
	LDD	F	*p*	Mean	SEM	Mean	SEM	t	Sig.	Mean	SEM	t	Sig.	Mean	SEM	t	Sig.
Feeding visits	6	7.75	0.0001	27.81	2.86	18.29	2.72	2.31	0.0494	32.10	3.42	-0.25	0.8031	41.00	3.13	-2.79	0.0189
	5	2.76	0.0458	42.33	1.94	37.50	1.72	1.70	0.0943	38.23	2.03	1.48	0.1453	35.17	3.09	2.52	0.0502
	4	7.30	0.0002	19.48	0.93	20.64	0.99	-0.79	0.4352	20.00	1.34	0.10	0.9187	14.66	1.02	3.58	0.0022
	3	7.34	0.0002	11.33	0.88	12.18	1.05	-0.55	0.5837	10.67	0.77	0.62	0.5369	7.48	0.61	3.62	0.0019
	2	12.78	0.0000	6.78	0.69	7.68	0.85	-0.62	0.5381	6.63	0.84	0.65	0.5187	2.97	0.23	6.03	0.0000
	1	4.19	0.0075	4.59	0.58	6.50	0.77	-1.78	0.0807	5.07	0.62	-0.43	0.6700	3.52	0.55	1.76	0.0844
Cumulative feeding visits	6	3.00	0.0336	112.33	1.83	102.79	2.82	2.88	0.0184	112.70	3.15	1.13	0.8599	104.79	3.17	2.17	0.0712
	5	7.18	0.0002	84.52	3.24	84.50	4.07	0.22	0.8279	80.60	4.61	1.76	0.2812	63.79	4.67	4.00	0.0008
	4	11.64	0.0000	42.19	2.16	47.00	2.72	-1.13	0.2656	42.37	2.97	1.65	0.6877	28.62	1.82	4.84	0.0000
	3	11.68	0.0000	22.70	1.48	26.36	2.04	-1.03	0.3061	22.37	1.81	1.69	0.5713	13.97	0.95	5.04	0.0000
	2	9.99	0.0000	11.37	1.07	14.18	1.39	-1.27	0.2090	11.70	1.30	1.60	0.7873	6.48	0.65	4.33	0.0002
	1	4.05	0.0090	4.59	0.58	6.50	0.77	-1.65	0.1058	5.07	0.62	0.74	0.7126	3.52	0.55	1.83	0.0731
Feeding duration	6	8.37	0.0000	38.09	3.88	25.64	3.67	2.35	0.0470	46.43	5.04	-0.28	0.7841	63.62	5.04	-3.27	0.0056
	5	1.26	0.2919	65.50	2.61	58.73	2.54			61.96	2.31			60.33	3.93		
	4	8.46	0.0000	41.22	2.07	43.53	1.75	-0.97	0.3362	41.34	2.45	0.26	0.7939	30.74	2.07	3.64	0.0018
	3	7.20	0.0002	22.26	1.40	23.91	1.69	-0.51	0.6101	21.92	1.88	0.67	0.5079	15.16	1.30	3.55	0.0024
	2	14.99	0.0000	11.86	1.14	13.68	1.63	-0.62	0.5389	11.42	1.45	0.89	0.3762	4.84	0.42	6.24	0.0000
	1	5.13	0.0023	7.84	0.93	11.86	1.81	-1.56	0.1246	8.37	1.10	0.27	0.7916	5.38	0.71	2.71	0.0273
Cumulative feeding duration	6	2.01	0.1169	186.77	3.06	177.35	4.50			191.43	4.79			180.08	4.70		
	5	7.64	0.0001	148.68	4.77	151.70	6.42	-0.15	0.8834	145.00	7.07	0.80	0.4302	116.45	7.02	4.02	0.0007
	4	12.54	0.0000	83.18	3.97	92.98	5.14	-1.23	0.2237	83.04	5.79	0.54	0.5903	56.12	3.58	5.08	0.0000
	3	12.20	0.0000	41.96	2.43	49.45	4.05	-1.01	0.3157	41.70	3.72	0.75	0.4552	25.38	1.78	5.35	0.0000
	2	11.59	0.0000	19.70	1.81	25.54	2.91	-1.27	0.2108	19.78	2.28	0.60	0.5530	10.22	0.89	5.12	0.0000
	1	5.13	0.0023	7.84	0.93	11.86	1.81	-1.56	0.1246	8.37	1.10	0.27	0.7916	5.38	0.71	2.71	0.0273

Supplementary Table S1. (continued)

June 2016

		ANOVA		C		C1				C10				T200			
				n = 6		n = 5				n = 14				n = 4			
	LDD	F	*p*	Mean	SEM	Mean	SEM	t	Sig.	Mean	SEM	t	Sig.	Mean	SEM	t	Sig.
Feeding visits	6	5.19	0.0063	23.17	8.44	28.60	9.02	-0.64	0.5366	51.64	3.55	-2.87	0.0800	35.50	2.96	-2.03	0.0918
	5	8.14	0.0006	49.67	7.95	59.60	6.75	-1.06	0.3149	32.29	2.38	2.95	0.0255	35.50	2.53	1.41	0.1973
	4	4.52	0.0115	24.50	4.58	22.20	2.85	0.11	0.9137	13.57	1.73	2.80	0.0353	15.25	1.03	1.37	0.2093
	3	2.98	0.0505	11.50	1.88	7.60	0.93			7.43	0.75			7.25	0.75		
	2	1.72	0.1887	6.33	2.17	3.00	0.55			3.93	0.46			3.50	0.50		
	1	1.23	0.3186	3.67	0.67	3.20	0.58			3.29	0.45			2.00	0.41		
Cumulative feeding visits	6	2.31	0.1012	118.83	9.98	124.20	9.29			112.14	2.49			99.00	3.76		
	5	6.72	0.0018	95.67	14.79	95.60	8.42	-0.23	0.8240	60.50	5.06	3.11	0.0180	63.50	1.89	2.22	0.0741
	4	3.81	0.0224	46.00	8.66	36.00	3.51	0.86	0.4122	28.21	2.80	2.83	0.0333	28.00	0.91	1.89	0.0959
	3	2.60	0.0748	21.50	4.48	13.80	1.77			14.64	1.28			12.75	0.75		
	2	1.59	0.2179	10.00	2.73	6.20	1.07			7.21	0.69			5.50	0.87		
	1	1.31	0.2925	3.67	0.67	3.20	0.58			3.29	0.45			2.00	0.41		
Feeding duration	6	5.84	0.0036	29.57	12.32	38.32	10.06	-1.03	0.3291	67.16	4.64	-2.74	0.1066	55.33	5.49	-2.43	0.0549
	5	5.76	0.0039	72.36	6.68	86.45	6.52	-1.60	0.1444	60.21	2.88	2.01	0.0602	66.38	4.13	0.61	0.5583
	4	3.34	0.0354	52.13	8.93	45.53	4.92	0.31	0.7667	32.58	2.94	2.54	0.0611	35.94	3.25	1.18	0.2723
	3	4.18	0.0158	26.28	3.68	16.57	1.52	2.63	0.0547	16.97	1.55	3.04	0.0213	15.68	1.94	2.69	0.0273
	2	1.35	0.2794	12.97	4.04	6.56	1.19			8.60	1.09			7.19	0.85		
	1	1.94	0.1488	9.15	1.89	5.93	1.38			7.32	0.89			4.47	1.01		
Cumulative feeding duration	6	0.63	0.6006	202.45	13.52	199.36	5.74			192.83	4.83			184.98	5.41		
	5	5.06	0.0071	172.88	19.92	161.04	9.96	0.35	0.7316	125.67	7.30	3.01	0.0228	129.65	2.35	2.30	0.0669
	4	4.05	0.0177	100.52	16.68	74.59	5.29	1.43	0.1863	65.46	5.16	2.95	0.0256	63.28	2.47	2.19	0.0602
	3	3.85	0.0215	48.39	9.05	29.06	3.32	2.31	0.0463	32.88	2.59	2.41	0.0810	27.34	2.06	2.46	0.0783
	2	2.07	0.1299	22.12	5.77	12.49	2.48			15.92	1.56			11.66	1.40		
	1	1.94	0.1488	9.15	1.89	5.93	1.38			7.32	0.89			4.47	1.01		

July 2016

		ANOVA		C		C1				C10				T200			
				n = 5		n = 5				n = 5				n = 5			
	LDD	F	*p*	Mean	SEM	Mean	SEM	t	Sig.	Mean	SEM	t	Sig.	Mean	SEM	t	Sig.
Feeding visits	6	2.25	0.1220	6.20	2.62	14.20	3.99			7.60	2.32			3.80	2.62		
	5	1.52	0.2478	49.40	3.08	46.60	3.91			51.20	2.29			56.60	3.97		
	4	3.00	0.0615	25.20	2.35	20.60	2.69			25.40	1.99			29.40	1.29		
	3	2.02	0.1511	12.40	0.68	9.80	1.28			11.40	0.24			12.20	0.97		
	2	7.08	0.0030	8.80	1.16	4.20	0.20	5.17	0.0026	5.60	0.98	2.23	0.0567	7.00	0.55	1.43	0.1904
	1	0.09	0.9643	5.20	0.97	4.40	0.68			5.00	1.10			4.80	0.58		
Cumulative feeding visits	6	2.69	0.0813	107.20	3.48	99.80	3.72			106.20	3.92			113.80	2.67		
	5	4.22	0.0223	101.00	2.45	85.60	6.59	2.09	0.0939	98.60	4.88	1.09	0.6349	110.00	4.09	-1.87	0.0981
	4	4.64	0.0162	51.60	3.71	39.00	3.61	2.44	0.1214	47.40	2.93	0.40	0.4153	53.40	1.50	-0.56	0.5937
	3	3.48	0.0406	26.40	2.42	18.40	1.44	2.68	0.0838	22.00	1.79	0.57	0.2181	24.00	1.00	0.73	0.4840
	2	2.23	0.1245	14.00	1.90	8.60	0.81			10.60	1.81			11.80	0.58		
	1	0.08	0.9697	5.20	0.97	4.40	0.68			5.00	1.10			4.80	0.58		
Feeding duration	6	2.37	0.1092	7.59	3.27	20.01	5.97			12.34	3.49			5.58	3.39		
	5	0.39	0.7628	70.45	3.45	74.71	5.79			75.12	3.38			80.80	9.69		
	4	3.14	0.0545	48.68	3.14	44.69	4.81			50.85	2.85			59.80	1.96		
	3	3.53	0.0391	29.90	1.03	22.64	2.58	2.31	0.0500	26.05	0.81	2.89	0.0609	28.73	1.42	0.71	0.4966
	2	8.79	0.0011	18.13	2.08	8.65	0.45	5.65	0.0014	10.45	1.63	2.83	0.0440	14.89	1.15	1.29	0.2347
	1	0.58	0.6342	10.53	1.66	7.45	0.73			9.74	2.38			9.93	1.27		
Cumulative feeding duration	6	2.46	0.1000	185.29	4.31	178.14	5.65			184.54	4.51			199.74	8.11		
	5	4.68	0.0157	177.70	3.55	158.13	8.26	2.12	0.0672	172.20	5.29	0.87	0.4078	194.15	8.92	-1.77	0.1150
	4	8.27	0.0015	107.25	5.10	83.42	6.21	2.93	0.0568	97.08	3.46	1.65	0.1366	113.35	1.97	-1.17	0.2738
	3	7.01	0.0032	58.56	4.02	38.73	2.83	3.79	0.0160	46.23	3.45	2.23	0.0565	53.55	1.81	1.02	0.3390
	2	4.06	0.0254	28.67	3.54	16.10	1.08	3.76	0.0167	20.18	3.66	1.63	0.1418	24.82	0.50	0.84	0.4450
	1	0.58	0.6342	10.53	1.66	7.45	0.73			9.74	2.38			9.93	1.27		

Supplementary Table S1. (continued)

		ANOVA		Mean: C (n = 4)		C1 (n = 4)		C10 (n = 4)		T200 (n = 4)	
	LDD	F	*p*	Mean	SEM	Mean	SEM	Mean	SEM	Mean	SEM
**Feeding visits**	6	0.18	0.9088	19.28	4.65	22.40	3.65	30.88	9.01	33.03	10.31
	5	1.16	0.3665	47.45	1.74	48.58	4.58	40.18	3.97	42.03	5.03
	4	0.91	0.4649	23.13	1.27	20.50	0.74	19.65	2.41	18.83	3.53
	3	1.2	0.3509	11.23	0.56	9.53	1.00	9.48	0.94	8.93	1.13
	2	1.97	0.1729	7.05	0.60	4.88	1.00	4.80	0.79	4.40	0.90
	1	1.18	0.3598	4.43	0.32	4.48	0.72	4.00	0.62	3.25	0.60
**Cumulative feeding visits**	6	0.13	0.9426	112.50	2.39	110.35	5.61	108.98	2.00	110.38	5.44
	5	1.43	0.2815	93.25	3.47	87.95	2.57	78.08	7.99	77.35	11.06
	4	1.42	0.2843	45.80	2.09	39.38	2.66	37.90	4.31	35.33	6.07
	3	1.42	0.2852	22.68	1.35	18.88	2.68	18.25	2.29	16.55	2.54
	2	1.46	0.2745	11.45	0.90	9.35	1.71	8.78	1.43	7.65	1.41
	1	1.18	0.3598	4.43	0.32	4.48	0.72	4.00	0.62	3.25	0.60
**Feeding duration**	6	0.30	0.8239	26.03	6.49	31.03	4.90	43.55	11.43	49.88	15.30
	5	0.78	0.5270	71.38	2.40	76.38	6.47	66.40	3.38	69.70	4.33
	4	0.82	0.5051	47.75	2.31	43.45	1.19	42.70	3.89	40.20	6.62
	3	1.00	0.4257	24.73	2.12	20.45	1.69	21.00	1.98	19.80	3.13
	2	2.02	0.1656	13.80	1.45	9.65	1.49	9.25	1.06	8.78	2.16
	1	1.23	0.3401	8.90	0.60	8.18	1.29	7.48	1.11	6.35	1.21
**Cumulative feeding duration**	6	0.19	0.9016	192.55	4.01	189.15	6.63	190.38	1.99	194.73	7.68
	5	1.15	0.3704	166.55	6.35	158.10	2.27	146.85	9.57	144.90	17.08
	4	1.36	0.3007	95.18	5.36	81.73	4.23	80.45	6.62	75.18	12.97
	3	1.44	0.2808	47.45	4.07	38.28	4.25	37.70	3.77	34.93	6.43
	2	1.7	0.2197	22.73	2.05	17.83	2.75	16.75	2.12	15.13	3.31
	1	1.23	0.3401	8.90	0.60	8.18	1.29	7.48	1.11	6.35	1.21
